# Eag and HERG potassium channels as novel therapeutic targets in cancer

**DOI:** 10.1186/1477-7819-8-113

**Published:** 2010-12-29

**Authors:** Viren Asher, Heidi Sowter, Robert Shaw, Anish Bali, Raheela Khan

**Affiliations:** 1 Research fellow, Department of Obstetrics and Gynaecology, School of Graduate Medicine and Health, Royal Derby Hospital, Uttoxeter road, Derby DE22 3DT, UK; 2 Lecturer, Biological and Forensics Sciences, University of Derby, Keldeston road, Derby DE22 1GB. UK; 3 Professor and Head, Department of Obstetrics and Gynaecology, School of Graduate Medicine and Health, Royal Derby Hospital, Uttoxeter road, Derby DE22 3DT. UK; 4 Consultant Gynaecological Oncologist, Department of Obstetrics and Gynaecology, Royal Derby Hospital, Uttoxeter road, Derby DE22 3NE; 5 Associate Professor, Department of Obstetrics and Gynaecology, School of Graduate Medicine and Health, Royal Derby Hospital, Uttoxeter road, Derby DE22 3DT. UK

## Abstract

Voltage gated potassium channels have been extensively studied in relation to cancer. In this review, we will focus on the role of two potassium channels, Ether à-go-go (Eag), Human ether à-go-go related gene (HERG), in cancer and their potential therapeutic utility in the treatment of cancer. Eag and HERG are expressed in cancers of various organs and have been implicated in cell cycle progression and proliferation of cancer cells. Inhibition of these channels has been shown to reduce proliferation both in vitro and vivo studies identifying potassium channel modulators as putative inhibitors of tumour progression. Eag channels in view of their restricted expression in normal tissue may emerge as novel tumour biomarkers.

## Introduction

Cancer is one of the major killers throughout the world. It is estimated that a total of 1,529,560 new cancer cases and 569,490 deaths from cancer will occur in the United States in 2010 [1]. There is increasing evidence that ion channels are involved in various processes characteristic of cancer cells such as uncontrolled cell proliferation, migration and survival in hypoxic conditions [2].

Ion channels are integral membrane proteins that mediate the transfer of ions through the hydrophobic lipid bilayer of the cell membrane. They play an important role in a variety of functions that range from nerve/muscle excitation [3], regulation of blood pressure [4], through to sperm motility and capacitation [5]. Potassium K^+^channels comprise the largest family of ion channels encoded by ~300 genes with phenotypic diversity generated through alternative splicing, variable association of (homo/heteromultimerisation) of channel subunits and posttranslational modifications. In normal cellular function, K^+ ^channels are the main determinants of a cell's resting membrane potential. K^+ ^channels have also been linked to cell volume control[6,7], cell cycle progression[8] and cardiac repolarisation[9].

In recent years, expression of several K^+ ^channel subtypes has been described in a plethora of malignancies. In particular the role of voltage gated K^+ ^channels in cancer, has been reviewed in several excellent publications [2,10,11]. This review will focus specifically on the Eag and HERG voltage gated K^+ ^channels with their potential therapeutic applications in cancer.

## Historical perspective

The *Eag *gene, present on locus 50 of the X chromosome of the fruitfly *Drosophila melanogaster*, is a mutant of the *Shaker *gene [12], so called since flies afflicted with this mutation exhibited slow, rhythmic shaking of the legs with minimal shaking of wings or abdomen on exposure to ether anaesthesia [13,14]. In a bid to find homologous *Eag *genes in *Drosophila *and mammals, a further two- *Elk *(Eag like gene) and *Erg *(Eag related gene) were discovered. All members of the Eag family have >85% DNA sequence homology [15]. The International Union of Basic and Clinical Pharmacology (IUPHAR) have classified the Eag family as shown in Table 1. [16]

**Table 1:** Members of the Eag family

Previous name	Official IUPHAR name	Human gene name
Eag1, KCNH1a, Eag1a, Eag1b	Kv 10.1	KCNH 1

Eag2, KCNH5	Kv 10.2	KCNH 5

HERG, erg1, hergb	Kv 11.1	KCNH 2

erg2	Kv 11.2	KCNH 6

erg3	Kv 11.3	KCNH 7

elk3, elk1	Kv 12.1	KCNH 8

elk2, BEC 1	Kv 12.2	KCNH 3

elk1, BEC 2	Kv 12.3	KCNH 4

Eag- ether à-go-go, HERG- Human ether à-go-go related gene, erg- ether à-go-go related gene, elk- ether à-go-go like, BEC- Brain Eag-like channel, KCNH- potassium channel H family.

The Eag channel has also been cloned from rat (rEag) [17], and bovine retina [18]. The first human Eag (hEag), located on chromosome 1q 32-41, was cloned from cultured myoblasts at the onset of fusion, but was absent in adult skeletal muscle, [19,20] indicating that expression of hEag is linked to the early stages of syncytial myotube formation.

The human HERG gene was the first member of the Ether-a go-go family to be isolated by screening of human hippocampal cDNA with the mouse homologue of Eag and was localised to chromosome 7 [15]. It has also been implicated in Long QT Syndrome 2 [21].

### Location and function of Eag and HERG

Eag channels are expressed in fusing myoblasts and been posulated to have a role in their hyperpolarisation that preceeds their fusion [19]. Eag channels are also selectively expressed in the brain and placenta of rat and humans [19,22], with diffuse immunohistochemical reactivity in rat brain. They are very noticeable in the perinuclear space of cells and proximal regions of the extensions, both in rat and human brain. The real time PCR analysis of rat brain revealed higher Eag 1 expression in olfactory bulb, cerebral cortex, striatum, hippocampus, hypothalamus, and cerebellum, and low expression in thalamus and brainstem [23].

The function of Eag channels in neurotransmitter release at the neuromuscular junctions to initiate action potential in *Drosophila melanogaster *larvae is well known [24] and recently genes for *shal *and *shaker *channels in the central nervous system of *Drosophila melanogaster *have been shown to be reciprocally regulated resulting in a target dependent, homeostatic modulation of synaptic transmission [25]. Eag channels are also involved in odour transduction [26] and are encoded in seizure locus in *Drosophila *[27]. In mammals, although Eag channels have been shown to be present in rat brain, their exact physiological function is not known, but in rat retina, they are known to be involved in the dark current-loop of photoreceptors. [28].

In contrast to Eag channels, HERG channels are more widely expressed and their functions differ according to their localization (Table 2). The HERG channel has a dominant presence in normal human myocardium where it is involved in the repolarisation phase of the cardiac action potential [21]. Mutations of this channel causes long QT syndrome 2 leading to cardiac arrhythmias and sudden death [29]. Gain of function mutations in this channel lead to short QT syndrome and sudden infant death [30].

**Table 2:** Location and function of HERG channel.

Organ	Function	References
Heart	Repolarisation of cardiac action potential	[21,29,30]

CNS	Maintain membrane potential and development of neurons of spinal cord and carotid glomus cells	[92,93]

GIT	Regulate motility of gut	[94]

Endocrine system	Secretion of insulin and modulating epinephrine release in chromaffin cells	[95,96]

## Structure of Eag channel family

Members of the Eag family share the same structure of other voltage-gated potassium channels, comprising of four identical α subunits each consisting of six membrane spanning domains (S1-S6) with cytoplasmic amino (N) and carboxy (C) termini. The ion-conduction pathway or pore region (P) is positioned between S5-S6, with voltage being sensed predominantly by the chain of positive arginine or lysine residues based at every third position, separated by two hydrophobic residues within the S4 domain which acts as a voltage sensor [31,32]. All the domains are well conserved among all the family members of Eag namely Eag, HERG and Elk, including the positively charged amino acids in the S4 segment [15]. The N terminal consists of a Per-Arnt-Sim (PAS) domain [33], a hypoxia sensor leading to the activation of hypoxia inducible factor (HIF1), resulting in increased glycolysis and angiogenesis thus conferring a selective growth advantage to cancer cells in a hypoxic environment [34,35]. The C terminus consists of a cyclic nucleotide binding domain (cNBD) and tetramerization-coil-coil domain with an Endoplasmic reticulum retention signal (RXR), which is involved in the tetramerization and functional expression of the channels [36,37]. Also present on the C terminus are multiple signalling modules including putative nuclear export sequences (NES) and nuclear localization sequences (NLS) with binding sites for calmodulin (CaM), calcium/CaM-dependent protein kinaseII (CaMKII) [38]. These NES and NLS play an important role in perinuclear localization of these channels.The structure of Eag channels is well conserved in *Drosophila*, mouse, rat and humans. The sequence comparisons among family members has shown that two members of the same subfamily in different species share about 60-70% amino acid identities from S1 through to the cNBD segment [39].

### Eag and HERG channels in cancer

The initial study reporting on a potential link between the Eag family of channels and cancer showed that high levels of *herg *mRNA were present in 17 cancer cell lines of different species (human and murine) with distinct histogenesis. These included neuroblastoma, rhabdomyosarcoma, adenocarcinoma, lung microcytoma, pituitary tumours, insulinoma B cells and monoblastic leukaemia [40].

Following this discovery, Walter Stuhmer's group showed that Chinese Hamster ovary (CHO) cells when transfected with rEag exhibited a transformed cancerous phenotype characterised by the ability of the cells to grow in a low concentration (0.5%) of serum, displaying increased DNA synthesis, higher metabolic activity and loss of contact inhibition [22]. The same group also demonstrated that *herg *mRNA is also expressed in MCF-7 (breast cancer), SHSY-5Y (neuroblastoma) and He-La (carcinoma cervix) cell lines. Inhibition by antisense oligonucleotides decreased the RNA content and functional protein of EF119 (breast cancer) cells [22]. Furthermore subcutaneous implantation of CHO cells expressing Eag channels in severe combined immune deficiency (SCID) mice lead to aggressive tumours showing intratumoral necrosis [22]. Eag channels also appear to impart a selective advantage for tumour cells in hypoxia by production of hypoxia inducible factor-1 (HIF-1) and thereby increasing vascular endothelial growth factor (VEGF) and increased vascularisation [35]. Additionally expression of Eag channels has been shown to be associated with re-organisation of the cytoskeleton and extracellular matrix thereby influencing adhesion, proliferation and metastasis of tumour cells [41]. These experiments collectively demonstrate the oncogenic potential of Eag channels and hence their activation in cancer cells.

Eag channels have also been shown to have increased expression in various cancer cell lines namely IGR1, IPC298, and IGR39 (melanoma) [42], SH-SY5Y (neuroblastoma) [43] and MCF-7 (breast cancer) cell lines and various cancers such as gliomas [44], cervical cancers [45], colon carcinoma [46], gastric cancers [47] and sarcomas [48].

HERG channels have been shown to be expressed in various cell lines like human and murine neuroblastoma, human leukaemia (preosteoclastic, lymphoblastic, myelogenous and promyelocytic) [49-51], human rhabdomyosarcoma, colon carcinoma, mammary carcinoma, squamous cervical, endometrial cancer, gastric and glioblastoma [52-55].

The first tissue expression of HERG channels in cancer showed that *herg *mRNA and HERG protein was expressed in 67 and 82% of endometrial cancer tissues compared to 18% of normal endometrium with no expression seen in endometrial hyperplasia [56]. The same group also showed that both *herg *gene and HERG protein were expressed in blast cells of acute myeloid leukaemia patients while no expression was seen in peripheral blood mononuclear cells [57]. Similar results were demonstrated in lymphocytic leukaemia with no HERG expression in normal lymphocytes [51]. Prolactinoma cells have been shown to express the *herg *transcript and HERG channels have been suggested to a play a role in prolactin secretion [58].

Further investigation of HERG channels in cancer invasion and metastasis revealed that, in addition to the high expression of *herg *gene and HERG protein in colorectal cancers, highest expression is seen in metastatic cancers with absence in normal colon and adenomas. HERG channels also modulate the invasiveness of colon cancer thought to be directly related to the amount of HERG protein present on the cell membrane [59] and confirmed by HERG expression in gastric [60] and melanoma cells [61]. Increasing expression is associated with high grade tumours, furthermore knocking down of *herg *gene by siRNA resulted in reduced proliferation and invasiveness of the cells. In contrast high grade gliomas have shown lower expression of *herg *gene compared to high grade tumours [44] while there is loss of HERG expression in renal cell cancer compared to normal kidney [62].

These studies show that Eag and HERG channels are expressed by a variety of cancer cell lines and tissues with Eag channel showing an oncogenic potential while HERG channels are associated with more aggressive tumours and have a role in mediating invasion.

### Eag in cancer prognosis

Eag has been shown to have high expression in colorectal cancers compared to adenomas and its expression correlates with tumour size, lymph node metastasis and Dukes staging suggesting its role as a prognostic marker [63]. Similar studies in gastric cancer have shown that higher Eag expression is associated with higher stage and lymph node metastasis, which are known poor prognostic markers [47]. Recently Eag has been shown to be present in acute myeloid leukaemia and the channel expression strongly correlated with increasing age, higher relapse rates and significantly shorter survival [64].

### Regulation of Eag channels in cancer

Eag channels have been found to be up regulated in mouse colon on treatment with chemical carcinogens such as Dimethylhydrazine (DMH) and *N*-methyl-*N*-nitrosourea (MNU) compared to chemically induced Dextran sulphate sodium (DSS) colitis. These carcinogens are well known to induce premalignant changes in the colon mucosa. Moreover there was higher Eag protein expression and mRNA in the distal mouse colon treated by DMH and MNU compared to the untreated proximal colon which suggests their role in pathogenesis of colon cancer [46]. Estrogen has also been shown to increase Eag expression by its action on Estrogen receptor α (ERα) in cervical and lung carcinoma cells [65]. The same group also showed that keratinocytes expressing HPV oncogene expressed Eag compared to its lack of expression in normal keratinocytes. Higher Eag expression was also demonstrated in cervical cancer cells containing high risk HPV16 and 18 [65]. Other factors that increased Eag expression and activity are Insulin like growth factor-1 (IGF1) in Breast (MCF-7) cells through the akt pathway [66] and Arachidonic acid (AA)in melanoma cells [67].

### Use of Eag expression as a potential tumour marker

The potential of using Eag channel expression as a tumour marker is supported by observation that Eag channels show higher expression in all patients with cervical cancer [45]. In the normal group, there was higher expression in patients with human papilloma virus (HPV) infection who had negative smears and other premalignant conditions such as atypical hyperplasia of endometrium and serous cystadenoma of ovary. Moreover one patient with a negative smear had an unexpected finding of endocervical adenocarcinoma with positive Eag expression at hysterectomy suggesting its role as tumour marker and a potential early predictor of cancer [45].

Injection of poly-lysine containing recombinant anti Eag1 antibody conjugated to Cy5.5 into immune deficient mice grafted with MBA-MB-435 S mammary cancer cell line clearly showed the tumour and the sentinel lymph node on near infrared fluorescent imaging (NIF) in 24 hours. [68].

The increased expression of Eag channel in the mouse colon as a result of DMH exposure has been shown to be associated with poor survival. Eag has also be shown to be present at premalignant stage in the development of colon cancer therefore Eag transcripts present in stool samples and rectal biopsies may be useful as diagnostic and prognostic markers [46]. Thus Eag could be potentially used as a tumour marker for various cancers.

The next question now arises: what is the role of these channels in proliferation and cell cycle and how are they associated with carcinogenesis? We have now started to get some answers but still are quite far away from determining their exact role in carcinogenesis

### Role of Eag and HERG channels in cell proliferation and the cell cycle

The indication of an erg like inward rectifier being involved in cell cycle came initially from neuroblastoma cells that showed current characteristics resembling those of erg channels with a rapid reduction in the current when the cells were synchronised in G0/G1 phase or G1/S boundary of the cell cycle [49]. This novel inward rectifier also maintained the resting membrane potential at a more negative value an important feature of cancer cells[49]. Subsequently a slow activating potassium channel current similar to rat Eag (rEag) in neuroblastoma cells (h-Eag) was characterised and it was demonstrated that the electrical current was reduced to 5% of the control value when the neuroblastoma cells were synchronised to G1 phase of the cell cycle on treatment of retinoic acid, thus indicating their role in cell cycle [43].

Xenopus oocytes are a useful model for the study of the cell cycle as they are indefinitely arrested in the G2 phase of the first meiotic cycle, until a hormonal stimulus, for example progesterone, induces progression of meiotic division. Rat Eag (rEag) channels expressed in Xenopus oocytes reduce their activity when their maturation is induced by progesterone and also by Mitosis promoting factor as the oocytes progress through the cell cycle, denoting that Eag channels are cell cycle sensitive [69]. The partial syncronization of Xenopus oocytes cells in G0/G1 or M phase greatly increased the block by intracellular sodium (Na^+^) and caesium [70] which may be due to interaction of Eag channels with microtubules which are depolymerised during cell cycle [71]. Human Eag (hEag) has been showed to be transiently expressed before myoblast fusion and contribute to the hyperpolarisation that drives the process. As myoblast fusion involves withdrawal from cell cycle to form skeletal muscle, Eag channels have been suggested to be involved in their cell cycle regulation [19].

The expression of *herg *gene is not detectable in peripheral blood mononuclear cells (PBMNC) and circulating CD34^+ ^cells, but then is rapidly expressed as soon as they enter S phase on upon treatment with cytokine/growth factor mixture, suggesting that HERG channels play a role in cell cycle regulation [57]. Subsequently an N-truncated herg1b isoform was shown to coexist with *herg1 *RNA in human myeloid leukaemias. Both HERG1 and HERG1b proteins were demonstrated on the plasma membranes and can form heterotetramers. The expression of these isoforms was found to oscillate during cell cycle, with HERG1 protein upregulated in G1 phase and down regulated in S phase, while the N truncated HERG1b isoform upregulated in S phase [52]accounting for the variations in HERG currents in the mitotic cycle as shown in neuroblastoma cells [49].

The Eag and HERG channels have been shown to be inhibited in tissues of varying histology by Eag and HERG blockers which are reviewed in [10,11,72]. Imipramine a known Eag blocker induces apoptosis in acute myeloid leukaemia cells via the caspase-3 activation [73] while it has been shown that HERG expressing cells are more sensitive to apoptosis induced by hydrogen peroxide, with reversal of effect on blocking with a HERG blocker dofetilide [74]. The same authors also showed co-expression of HERG and TNFα on cell membrane of tumour cells, leading to increased activity of the transcription factor nuclear factor kappa B facilitating tumour cell proliferation [74]. Thus both Eag and HERG channels are associated with cell proliferation and play an important role in modulation of cell cycle.

### Hypothetical model of potential oncogenic mechanisms

**Figure 1:**
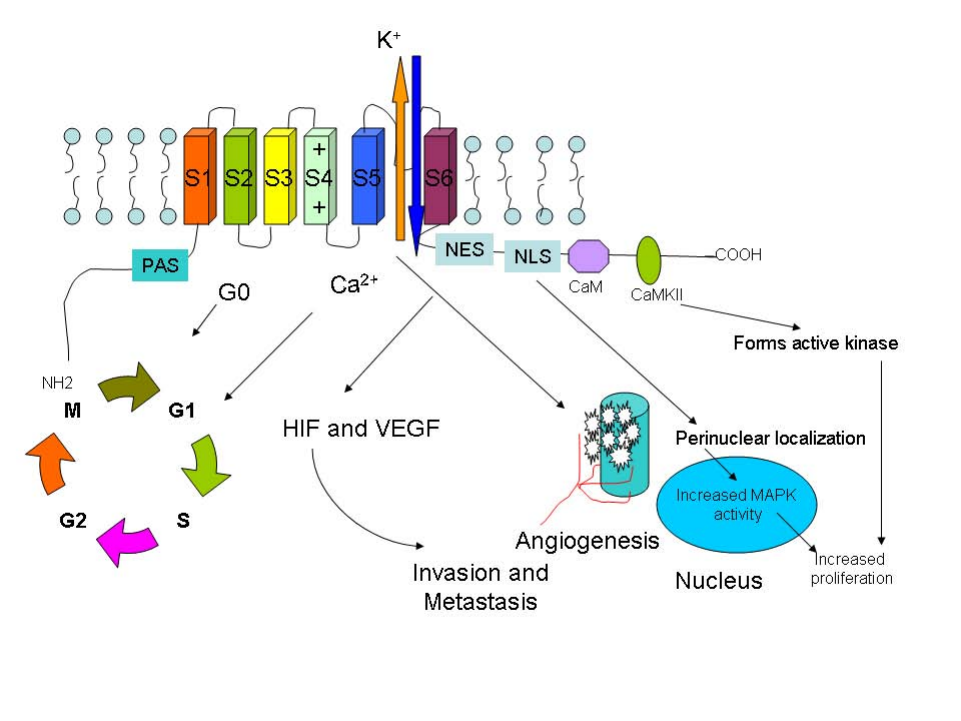
**Potential mechanisms of malignant transformation by K^+ ^channels**. Increased expression of K^+ ^channels on cell membrane results in increased influx of Ca^2+ ^ions resulting in increased transition of cells through G1/S phase of cell cycle. The channels in presence of hypoxia lead to release of HIF1 and VGEF factor leading to increased angiogenesis and subsequent invasion and metastasis of tumours. The nuclear localisation sequence (NLS) in the C terminus on activation results in perinuclear localisation of the channel leading to activation of Mitogen activated protein kinase (MARP) pathway resulting in increased cell proliferation. The Eag channels also act through the Ca calmodulin pathway to activate cell proliferation.

(Summarised in Figure 1)

As we have discussed, there is considerable evidence to support a role for Eag and HERG channels in cancer. However it is not at all clear whether these channels play causal roles in oncogenesis or whether the oncogenic process results in aberrant expression and activation of the Eag channel family. Identifying the mechanism underlying malignant transformation involving Eag channels especially is further compounded by a lack of specific pharmacological agents. Despite these, several theories have been advanced as to how Eag and HERG channels could promote malignant transformation as discussed below:

It is well known that K^+ ^channels play an important role in regulation of membrane potential in both excitable and non excitable cells. Nilius et al.[75], proposed that in human melanoma cells overexpression of K+ channels leads to hyperpolarisation as a result of the efflux of cations from the cell interior, which subsequently causes inward movement of Ca^2+ ^ions to maintain the membrane potential. The role of Ca^2+ ^in the transition from the G1 to the S phase during mitosis in mammalian cells is well-documented and Ca^2+ ^acts as a pacemaker that initiates the timing of cell cycle transitions [76]. Therefore increased intracellular Ca^2+ ^can trigger the rapid transition of cells through the G1 to S phase leading to enhanced proliferation. However the pathway through which this Ca^2+ ^entry occurs is not known and change in resting potential is not always observed when the cells are inhibited by potassium channel blockers [77].

An alternative mechanism postulated concerns the inverse relationship between cell volume and K^+ ^channels. Increasing K^+ ^channel activity leads to cell shrinkage which then deforms the cell and modifies the cytoskeletal components through changes in protein kinases or phosphatases that control cell proliferation [78]. This hypothesis is supported by the fact that K^+ ^channel blockers lead to an increase in cell volume and inhibit proliferation. However, it is argued that astrocytes that are involved in the formation of the blood brain barrier [79], despite having high expression of K^+ ^channels undergo a reduction in cell volume in presence of K^+ ^channel blockers while L-glutamate initiated K^+ ^influx into the cell leads to their swelling [80].

Hypoxia has been implicated as a stimulus for rapidly growing tumours where, hypoxic areas lead to altered cellular mechanisms consequently causing either an increase in oxygen or activation of other mechanisms not requiring oxygen. The induction of hypoxia inducible factor (HIF-1α) by hypoxia, subsequently leads to the transcriptional activation of genes encoding erythropoietin, VEGF and glycolytic enzymes, all thought to be involved in various aspects of tumour initiation, growth and metastasis [81]. HEK cells transfected with Eag channels lead to increased production of HIF-α in under hypoxic conditions and as Eag channels are overexpressed in various cancers, they could potentially confer selective advantage to cancer cells in hypoxic conditions [35].

There has been increasing evidence linking mutant Eag channels that contain non conducting subunits lacking functional pore with cell proliferation. Hegle et al [82] demonstrated the voltage dependent gating of the Eag channel controlled the cell proliferation and Mitogen activated protein kinase (MAPK) signalling pathway by a mechanism that is independent of K^+ ^influx through the channel. Eag channels also act as a scaffold for and activate Calcium -Calmodulin activated kinase II (CaMKII), forming a complex which remains active even in the presence of low calcium [83], leading to dysregulation of cell proliferation and apoptosis resulting in genesis of cancer [84].

Activated Nuclear localization sequence (NLS) located on the C terminus of Eag channels results in activation of Mitogen activated protein kinase (MAPK) signal transduction pathway that regulates cell morphology [38]. Sarcoma and cervical cells [48,65] have been shown to have increased perinuclear localization of Eag channels and NLS may play an important role in its oncogenic mechanism.

## Therapeutic application

From the above studies it is clear that blocking Eag and HERG channels inhibits cell proliferation and therefore disease progression. These channels have been demonstrated in the cell membrane by functional studies and therefore are accessible targets for modulation by drugs. Moreover Eag channels have restricted expression in the central nervous system, placenta and in myoblasts just prior to fusion but are expressed in cancer cell lines of various origin and cancer tissues making them a potential marker and target for various drugs [10,19,22,85,86].

Both Eag and HERG belong to the same family of voltage gated K^+ ^channels and share 47% of the amino acid sequence [15]. Thus any drug acting on Eag channel may also block HERG channels leading to prolonged QT syndrome, cardiac arrhythmias and sudden death [21,29]. Therefore there is a need for specific targeted blockers for maximal inhibitory effect and reduction in side effects.

### Several approaches have been used to target or inhibit Eag channels in cancer

1. Chemical blockers: Imipramine and astemizole have been shown to abolish Eag currents and inhibit the cell proliferation of tumour cells and are easily available in the market for use [87,88]. However both these drugs have undesirable cardiovascular side effects due to HERG blockade which limits their applicability in treating cancer.

2. Monoclonal antibodies: These act as highly specific molecules for a targeted blockade of the channels and minimise the side effects associated with action on homologous channels. These antibodies may also be potentially used as vehicles for therapeutic agents for a site specific action [86]. A monoclonal antibody has been designed against Eag1 with no effect on Eag2 and HERG channels. This antibody has been shown to reduce the K^+ ^channel currents in isolated cells and also inhibit the growth of cancer cells from various organs both in vitro and vivo. Hence evidence in favour of this antibody may potentially be used either alone or in association with current established treatment to reduce the dose and associated side effects of conventional chemotherapeutic drugs [89].

3. Inhibition of cell growth using small interfering RNA (si RNA) technologies: This is a potential new approach to knock down gene expression and reduce the amount of protein that is produced. The activity of Eag has been shown to be silenced by the use of Eag specific siRNA which result in reduced protein expression and inhibition of cell proliferation in various cancer cell lines with minimal non-specific side effects [90]. The challenge with this approach is the design of an appropriate transport vehicle and delivery of siRNA to the target organ and currently the subject of intense research.

### Targeting HERG channels

1. Short hairpin (sh) RNA technology: The knock down of *herg *gene expression by the use of shRNAs for HERG1 and the HERG-1b isoform, reduced growth rate, cell viability and inhibited colony formation of neuroblastoma cells restricting them to G0/G1 phase of cell cycle. There was also inhibition of tumour cells injected into nude mice on treatment with sh RNA. Thus this technology can be potentially used in silencing of *herg *gene and subsequently the reduction in growth of tumour, but its effect on the heart needs to be evaluated and the delivery of these molecules to target organs still poses a significant challenge [91].

2. Use of HERG blockers including E-4031 and ergtoxin have still not been tested in vivo studies but do show a promising role in potential use with chemotherapeutic agents or in chemoresistant disease. However tight cardiac monitoring will be needed due to the development of drug induced Long QT syndrome.

## Conclusion

Both Eag and HERG channels have been shown to be present in cancers of differing origin and have a role in cell proliferation, progression and survival. There is abundant data on the effects of various blockers on the inhibition of cell growth and these channels may prove to be promising novel therapeutic targets for the treatment for cancer. They can be potentially be used in conjunction with chemotherapeutic agents or can be used in chemoresistant disease to improve survival. Eag due to its restricted expression shows a promising role as a potential tumour marker.

## Competing interests

The authors declare that they have no competing interests.

## Authors' contributions

VA wrote the manuscript, RK and HS conceptualised the project and helped in preparation of manuscript, RS and AB corrected the manuscript.
